# Weather- and climate-driven power supply and demand time series for power and energy system analyses

**DOI:** 10.1038/s41597-024-04129-8

**Published:** 2024-12-04

**Authors:** Enrico G. A. Antonini, Alice Di Bella, Iacopo Savelli, Laurent Drouet, Massimo Tavoni

**Affiliations:** 1 CMCC Foundation - Euro-Mediterranean Center on Climate Change, Lecce, Puglia Italy; 2 RFF-CMCC European Institute on Economics and the Environment, Milan, Lombardy Italy; 3 Politecnico di Milano, Milan, Lombardy Italy; 4 Università Bocconi, Milan, Lombardy Italy

**Keywords:** Energy science and technology, Climate sciences

## Abstract

Reaching net-zero carbon emissions requires large shares of intermittent renewable energy and the electrification of end-use consumption, such as heating, making the future energy system highly dependent on weather variability and climate change. Weather exhibits fluctuations on temporal scales ranging from sub-hourly to yearly while climate variations occur on decadal scales. To investigate the intricate interplay between weather patterns, climate variations, and power systems, we developed a database of time series of wind and solar power generation, hydropower inflow, heating and cooling demand using an internally consistent modeling framework. Here we focused on the European continent and generated country level time series extending between 1940 and 2100. Our database can be used for analyses aimed at understanding and addressing the challenges posed by the evolving energy landscape in the face of deep decarbonization and climate change.

## Background & Summary

A wider use of low-carbon energy sources and the electrification of end uses are expected to exacerbate the exposure of national power systems to meteorological variability and climate change. Intermittent low-carbon energy sources, such as wind and solar, are in fact highly variable in space and time and not always available in quantities needed to meet electricity demand^1,2^. The unreliability of solar and, to a larger extent, wind can be a serious issue when electricity systems are largely reliant on these energy sources. Reliable power systems based primarily on variable energy sources require effective grid management, backup power systems, and energy storage capacity^3–5^. This is especially true since rising temperatures and more frequent extreme events induced by climate change will pose new challenges to maintaining system reliability. Climate change-induced variations in temperature will strongly affect electricity demand in residential, commercial, industrial, and agricultural sectors, which in turn will require additional investments in peak generation capacity^6^. Also, the potential of other technologies to firm up the generation from intermittent renewables will be affected by an increased climate risk due to changes in precipitation, river flows and temperature. For example, thermoelectric power derived from nuclear energy or fossil fuels with carbon capture and storage will be affected by the combined impacts of lower summer river flows and higher river water temperatures^7^ Hydropower generation potential will be affected by shifts in evapotranspiration rates, precipitation, and snowpack amounts and melt timing^8^. Wind and solar generations may also be affected but little consensus has emerged on temporal and spatial variability in wind and solar resource changes under climate change^9^.

The planning of highly reliable energy systems requires the use of data with high temporal and spatial resolution to capture the effect of variable weather and changing climate^10^. Some energy datasets built from meteorological variables are already available. For example, the Copernicus climate change service (C3S) operational energy dataset provides climate and energy indicators for the European energy sector^11,12^. The C3S dataset is built from the ERA5 reanalysis data (the fifth generation reanalysis developed by the European Centre for Medium-Range Weather Forecasts)^13^, for the period from 1979 to present and climate projections of the EURO-CORDEX (the European branch of the international Coordinated Regional Climate Downscaling Experiment (CORDEX) initiative)^14^, for the period from 2005 to 2100. The C3S dataset includes time series of wind and solar power capacity factors, hydropower generation, and total electricity generation. Other examples are Renewable Ninja^15,16^, and Demand Ninja^17^, which provide wind and solar power, cooling and heating demand time series at hourly resolution for many countries and subregions using the historical climate data (1980 to 2019) of the Modern-Era Retrospective analysis for Research and Applications, Version 2 (MERRA-2)^18^. Hourly wind and solar capacity factor time series were also developed for historical (1950–2020) and near-future (2020–2050) periods by Bloomfield *et al*.^19^ from ERA5 reanalysis data. Their near future variables were estimated with reanalysis data that were climate-adjusted with projections from the PRIMAVERA project^20^. Another example is SECURES-Met^21^, which provides meteorological variables obtained from historical ERA5 reanalysis data (1981–2020) and climate projections (1951–2100) from one climate model of the EURO-CORDEX. The variables included in SECURES-Met are temperature, solar radiation, wind capacity factor, and hydropower potential aggregated for European countries and sub-regions. Lastly, Buster *et al*.^22^ obtained high resolution wind speeds, solar radiation, and temperature using lower resolution global climate model projections downscaled with generative machine learning, demonstrating the potential of these techniques in speeding up computational intensive downscaling processes. Although the availability of energy datasets is increasing, the availability within the same dataset of both historical and future data of weather- and climate-driven power supply and demand variables is missing.

Here, we present a database of time series of wind and solar power generation, hydropower inflow, heating demand, and cooling demand developed using an internally consistent modeling framework that uses both historical and projected meteorological variables. Our contribution is the estimation of these five energy variables with a set of logically aligned and uniformly applied assumptions and methods, as described in the following sections. Differently from previous studies (see Supplementary Table 1 for comparison), our dataset includes time series of European countries for any period between 1940 and 2100 and allows to capture the main impacts of weather and climate on the energy systems both on the supply and demand sides. Here, we focus on the European continent and apply the modeling framework to EU27 countries, Balkan countries, the United Kingdom, Norway, and Switzerland. The framework is however open-source, customizable, and applicable to any country of the world, with the meteorological database of choice (with different spatial and temporal resolution), and where appropriate energy infrastructure data are available. Here, we use both historical meteorological data from the ERA5 reanalysis and projected meteorological variables from three climate models of the EURO-CORDEX and three representative concentration pathways (trajectories of greenhouse gas concentration). On the one hand, the use of historical meteorological data allows the resulting time series to be used for analysis where actual climatic conditions are important such as assessing the potential of renewable energy or resource adequacy. On the other hand, the use of multiple projected meteorological variables from different models and scenarios allows the resulting time series to be used for analysis where deep uncertainties characterizing climate, policy and technological progress for emissions reduction are present. Lastly, the resulting time series, with the exception of hydropower inflow, are normalized to allow them to be incorporated in other modeling frameworks (e.g., power system models) where the quantities of the different components of power supply and demand (e.g., wind and solar capacities or heat pump adoption) can be individually chosen. The availability of our dataset aligns with recent findings that demonstrate the importance of incorporating extensive weather data—much more than is the current practice—in energy system planning to ensure reliability, particularly in capturing the variability and extreme events crucial for deeply decarbonized energy systems^23^.

## Methods

**Fig. 1:**
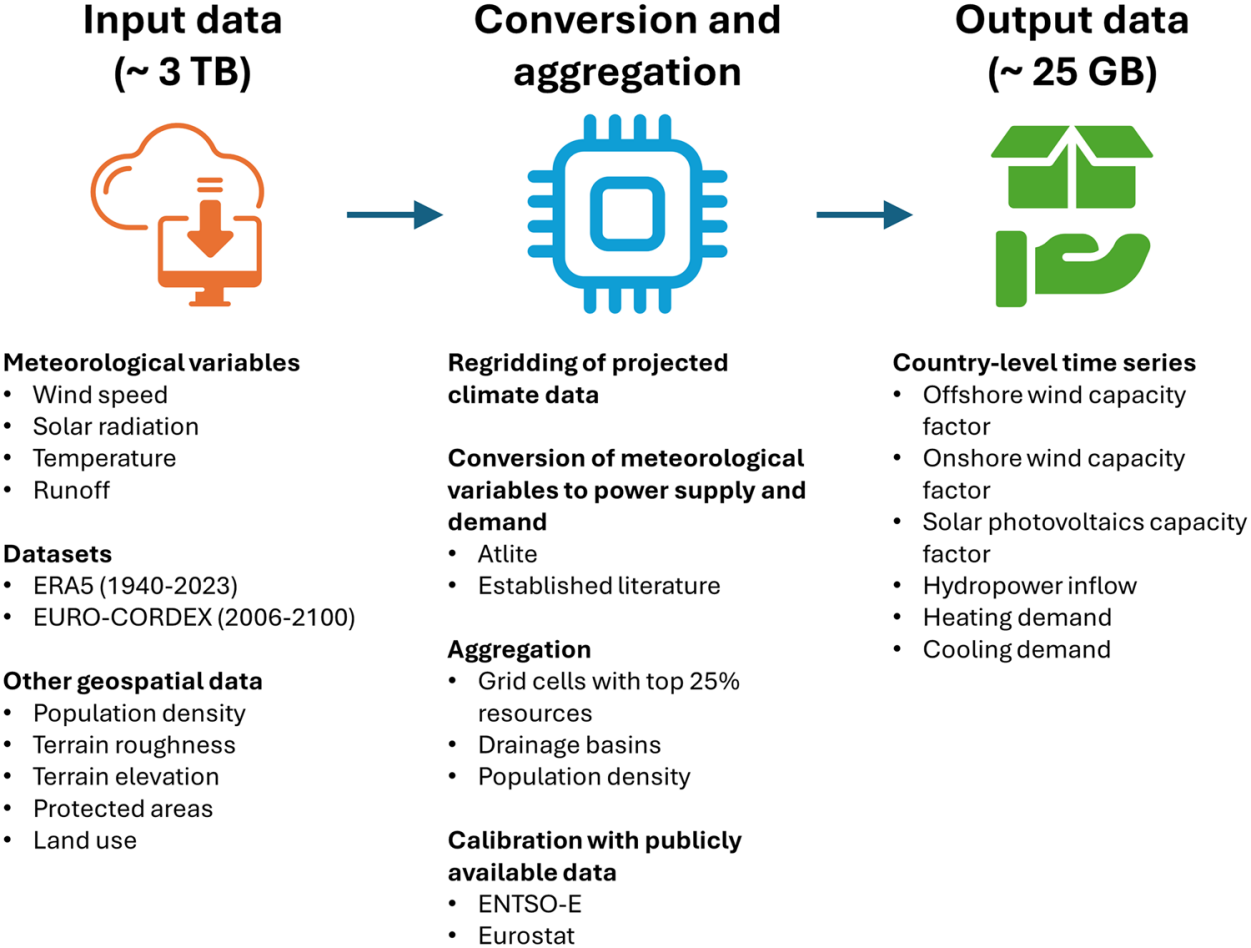
Workflow to estimate country-level time series of wind capacity factor, solar capacity factor, hydropower inflow, heating demand, and cooling demand. Meteorological variables such as wind speed, solar radiation, temperature, and runoff are retrieved from ERA5 reanalysis and EURO-CORDEX projections. Together with other geospatial data such as population density, terrain roughness and elevation, protected areas, and land use, we perform a conversion from meteorological to power variables using Atlite and methods from established literature. Lastly, we perform an aggregation to obtain country-level time series of offshore and onshore wind capacity factors, solar photovoltaic capacity factors, hydropower inflows, and heating and cooling demands.

**Fig. 2:**
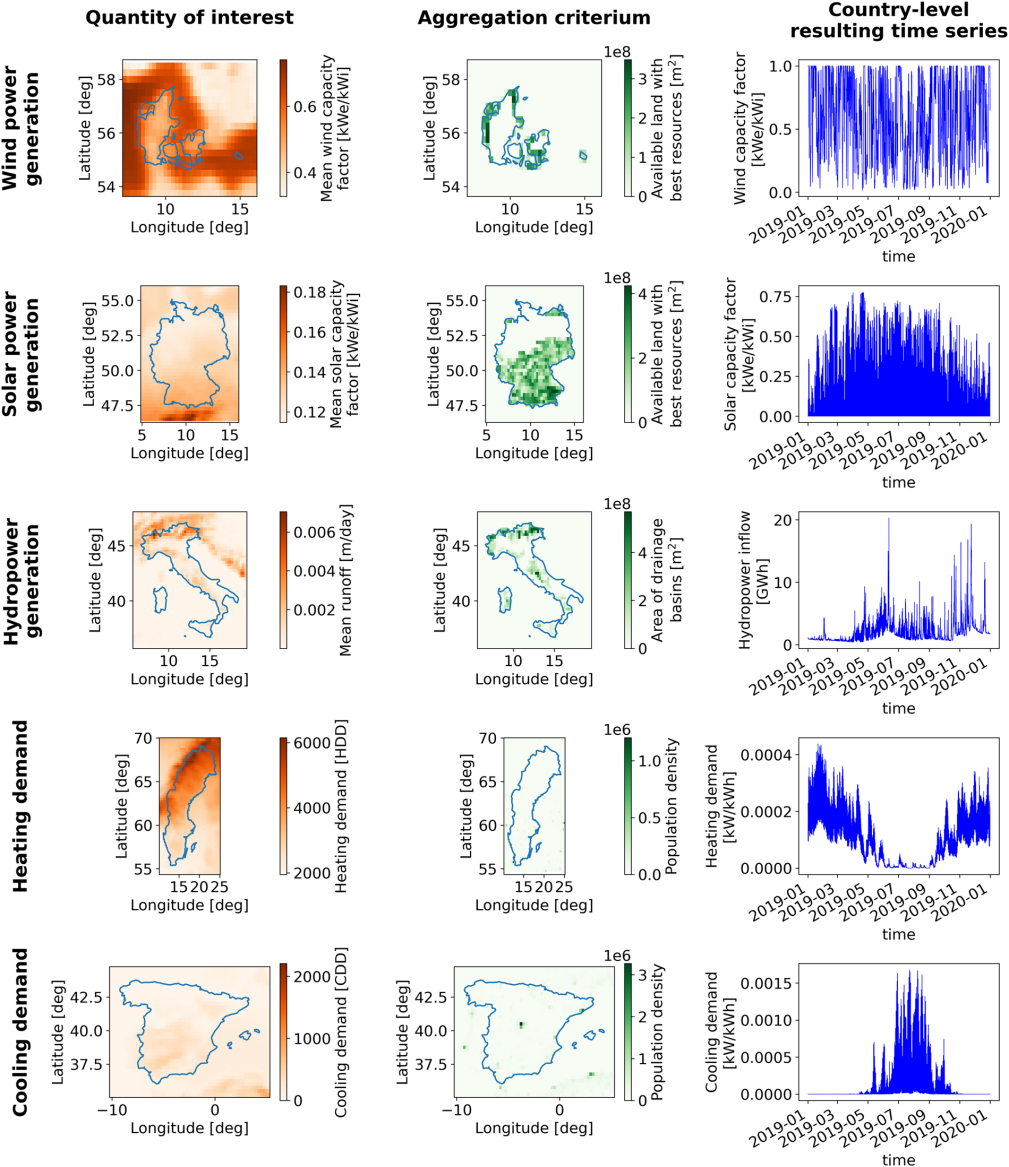
Example of variable aggregation and time series generation. The five examples consider wind power generation in Denmark, solar photovoltaic generation in Germany, conventional and pumped-storage hydropower generation in Italy, heating demand in Sweden, and cooling demand in Spain. Panels on the left show the grid-level mean value of the quantity of interest, panels in the middle show the gridded value of the variable used for aggregation, and panels on the right show the resulting country-level time series.

In this section, we present the methodology developed to estimate country-level time series of weather and climate-driven power supply and demand. An illustration of its workflow is presented in Fig. 1. In our methodology, we use several meteorological variables retrieved from the ERA5 and EURO-CORDEX datasets. The meteorological variables include wind speed, various components of the solar radiation, runoff, and temperature. A list of the meteorological datasets and variables used in the present paper is presented in Tables 1, 2. We gather other geospatial data, namely, protected areas from the World Database on Protected Areas (WDPA)^24^, land use from the Coordination of Information on the Environment (CORINE) dataset^25^, terrain roughness and elevation from the ERA5 dataset, and population density produced by the European Commission’s Joint Research Centre (JRC)^26^. From the meteorological variables, we perform a conversion to energy variables using Atlite^27^, and methods from the established literature, which will be explained in detail in the following sections. From the energy variables, we perform an aggregation using available land, drainage basins, or population density as illustrated in Fig. 2 and explained in detail in the following sections. Lastly, we perform a calibration of the time series of the power supply against generation data retrieved from the European Network of Transmission System Operators (ENTSO-E), which is explained in the technical validation section.

**Table 1:** Meteorological datasets used as input for generation and demand time series.

Historical data	Dataset	
	ERA5	
	Global climate model	Regional climate model
Future projections	CNRM-CERFACS-CM5	CNRM-ALADIN63
	MPI-M-MPI-ESM-LR	ICTP-RegCM4-6
	MIROC-MIROC5	CLMcom-CLM-CCLM4-8-17

We retrieve historical meteorological variables from the ERA5 reanalysis and projected variables from three combinations of global-regional climate models and three representative concentration pathways.

**Table 2:** Meteorological variables retrieved from the ERA5 and EURO-CORDEX datasets.

	Variable long name	Variable short name	Unit	Temporal resolution
ERA5	2-m temperature	t2m	K	1 hour
	100-m u-component of wind	u100	m s^−1^	1 hour
	100-m v-component of wind	v100	m s^−1^	1 hour
	Forecast surface roughness	fsr	m	1 hour
	Total sky direct solar radiation at surface	fdir	J m^−2^	1 hour
	Surface net solar radiation	ssr	J m^−2^	1 hour
	Surface solar radiation downwards	ssrd	J m^−2^	1 hour
	TOA incident solar radiation	tisr	J m^−2^	1 hour
	Runoff	ro	m	1 hour
EURO-CORDEX	2-m air temperature	tas	K	3 hours
	10-m wind speed	sfcWind	m s^−1^	3 hours
	Surface solar radiation downwards	rsds	W m^−2^	3 hours
	Surface upwelling shortwave radiation	rsus	W m^−2^	3 hours
	Total run off flux	mrro	kg m^−2^ s^−1^	6 hours

We report the variable long and short names, their unit, and their temporal resolution.

### Meteorological datasets

ERA5 is produced by the Copernicus Climate Change Service (C3S) at the European Centre for Medium-Range Weather Forecasts (ECMWF). ERA5 is the fifth generation ECMWF atmospheric reanalysis of the global climate covering the period from January 1940 to the present. It provides hourly estimates of a large number of atmospheric, land and oceanic meteorological variables. The data covers the Earth on a regular latitude-longitude grid of 0.25 degrees (about 30 km in the mid-latitudes) and 137 levels from the surface up to a height of 80 km. The next generation reanalysis product, ERA6, is under development as of 2024, and it will be a coupled reanalysis, using both atmospheric and ocean observations. When available, it can be used to update our time series.

EURO-CORDEX is the European branch of the international Coordinated Regional Climate Downscaling Experiment (CORDEX) initiative, which is a program sponsored by the World Climate Research Program (WRCP) to organize an internationally coordinated framework to produce improved regional climate change projections for all land regions world-wide. These projections are obtained by downscaling the Coupled Model Intercomparison Project Phase 5 (CMIP5) simulation results produced in support of the Intergovernmental Panel on Climate Change (IPCC) Fifth Assessment Report (AR5)^28^. Ensembles of CORDEX climate projections are available for different Representative Concentration Pathways (RCP) forcing scenarios. These scenarios are the RCP 2.6, 4.5 and 8.5 scenarios providing different pathways of the future climate forcing. The climate forcing quantifies the change in energy balance in the Earth’s atmosphere due to a carbon emission scenario (the higher the emissions, the higher the climate forcing, the higher the temperature). The data is on single levels on a regular latitude-longitude grid of 0.11 degrees. The period covered is 2006–2100 while the temporal resolution can be 3-, 6-hourly, daily, or monthly, depending on the variable. Note that we performed a linear interpolation between the data points to upsample the time series to hourly resolution consistent with historical data. The next generation of projections obtained by downscaling the CMIP6 simulation results are under development as of 2024, and when available, they can be used to update our time series. Based on data availability and consistency, our selected global climate models are CNRM-CERFACS-CM5^29^. MPI-M-MPI-ESM-LR^30^, and MIROC-MIROC5^31^. These three models respectively provide the boundary conditions for the regional climate models again selected based on data availability and consistency, which are CNRM-ALADIN63^32^ ICTP-RegCM4-6^33^ and CLMcom-CLM-CCLM4-8-17^34^. The use of multiple projected meteorological variables from different models and scenarios allows this database to be used for analysis where deep uncertainties characterizing climate, policy and technological progress for emissions reduction are present. Note that both ERA5 and EURO-CORDEX data may exhibit biases due to spatial and temporal resolution, terrain representation, and model parameterizations, leading to potential inaccuracies in capturing localized weather phenomena and extreme events^35^.

### Wind power generation

For each grid cell of the selected dataset, we calculate the time series of the wind power generation. This depends on the wind speed at the turbine hub height and the chosen turbine, which is characterized by a power curve. Here, we consider two wind turbines, the IEA-3.4-130-RWT^36^ for onshore and IEA-10.0-198-RWT^37^ for offshore installations. These wind turbines were developed within the IEA Wind Task 37^38^ The calculation of the wind power generation is summarized as follows, while full details can be found in the Supplementary Information: first, we calculate the wind speed (Eq. 1); second, the wind speed of the selected dataset at the given height is extrapolated to the turbine hub height using a well-established logarithmic function (Eq. 2); lastly, the resulting wind speed is used to get the power generation from the wind turbine power curve (Eq. 3). The extrapolation and conversion operations are performed using Atlite’s built-in functionalities.

To calculate the country-level time series of the wind capacity factor, we perform an aggregation using the available area with the wind power in the first quartile. For onshore applications, from the country’s available land, we subtract all protected areas of the World Database on Protected Areas (WDPA), as well as areas within 500 m from urban, industrial, and commercial centers. For offshore applications, from the country’s exclusive economic zone, we remove all protected areas and consider only water within 100 km from the coast. While we exclude all protected areas in this analysis, note that construction in areas with low protection status may still be possible under certain circumstances that vary on a case-by-case basis^39^. Of the resulting available area, we consider only the fraction with the resources in the first quartile. This area for potential installations allows room for other socio-economic constraints^40^, which are not considered in this methodology, while preserving more diversified wind resources. We consider the area for potential installations because currently installed capacity is still a marginal fraction in the supply mix and because locations of installed capacity are not always available.

### Solar photovoltaic power generation

For each grid cell of the selected dataset, we calculate the time series of the solar photovoltaic power generation. This depends on the time of the day, position on the Earth, solar irradiance, and panel efficiency. The calculation of the solar photovoltaic power generation is summarized as follows, while full details can be found in the Supplementary Information: first, we calculate the solar coordinates, i.e., the Sun’s altitude and azimuth (Eq. 4–14); second, given the solar position, we calculate the angle of incidence of the solar radiation on a tilted surface (Eq. 15); third, we calculate the total radiation received by a tilted surface, which is composed of direct, diffuse, and reflected radiation (Eq. 16-17); lastly, the solar photovoltaic power generation is calculated from the solar panel power curve, which depends on the incident solar radiation and ambient temperature (Eq. 18). The calculation of the Sun’s position, the incidence angle, the incidence radiation, and the power conversion are performed using Atlite’s built-in functionalities.

To calculate the country-level time series of the solar photovoltaic capacity factor, we perform an aggregation using the available area with the resources in the first quartile, in a way similar to the wind power capacity factor. To calculate the available land, we subtract all protected areas of the World Database on Protected Areas and terrain where its slope is larger than 10%, according to standard practice.

### Hydropower inflow

For each hydropower plant and for each dataset, we calculate the time series of the inflow following the methodology developed by Gøtske and Victoria^41^. The calculation of the hydropower inflow is summarized as follows, while full details can be found in the Supplementary Information: first, from the location of the currently installed hydropower plants (JRC Hydropower Database^42^) and hydrological maps (HydroBASINS^43^ with level 8 basins delineation), we identify the grid cells that belong to each plant’s upstream drainage basins; second, to determine the basin inflow, we aggregate runoff values in each basin and multiply the resulting value by its basin surface and water density (if ERA5 data are used) or time step resolution (if CORDEX data are used) (Eq. 19); third, using Atlite’s built-in functionality, the inflow to any hydropower power plant is calculated by considering the water flowing from any upstream basin to its reservoir (with an assumed speed equal to 1 m/s) and aggregating their values (Eq. 20); lastly, we estimate the hydropower inflow in unit of energy (Eq. 21). The country-level hydropower inflow is obtained by summing up the inflow of all hydropower plants.

### Heating demand

For each country and for each dataset, we calculate the time series of the space heating demand. This depends on the heating degree days, the intraday heating demand, and the population density. The calculation of the heating demand is summarized as follows, while full details can be found in the Supplementary Information: first, from the hourly time series of the temperature in each grid cell, we calculate using Atlite’s built-in functionality the heating degree days, i.e., the difference between the daily mean air temperature and the lowest daily mean air temperature not leading to indoor heating (Eq. 22); second, we aggregate the grid-cell-level time series of the heating degree days by performing a weighted average using the population density in each grid cell to obtain a country-level time series; third, we calculate normalized intraday hourly heating demand profiles, i.e., the heating demand over 24 hours, following the method developed by Ruhnau *et al*.^44^; fourth, we build an hourly heating demand time series by concatenating these intraday profiles multiplied by the respective heating degree days^44,45^; lastly, the time series is normalized by its time integral, such that the actual heating demand time series can be obtained by multiplying the normalized time series by the total annual heating demand retrieved from EUROSTAT data^46^.

### Cooling demand

For each country and for each dataset, we calculate the time series of the space cooling demand. This depends on the cooling degree days, the intraday cooling demand, and the population density. The calculation of the cooling demand is summarized as follows, while full details can be found in the Supplementary Information: first, from the hourly time series of the temperature in each grid cell, we calculate using Atlite’s built-in functionality the cooling degree days, i.e., the difference between the daily mean air temperature and the highest daily mean air temperature not leading to indoor cooling (Eq. 23); second, we aggregate the grid-cell-level time series of the cooling degree days by performing a weighted average using the population density in each grid cell to obtain a country-level time series; third, to get an intraday cooling demand profile, we calculate using a modified Atlite’s built-in functionality the cooling degree hours, i.e., the difference between the hourly air temperature and the highest hourly air temperature not leading to indoor cooling (Eq. 24); fourth, we keep the values of the cooling degree hours only on the days where cooling degree hours are positive; lastly, the time series is normalized by its time integral, such that the actual cooling demand time series can be obtained by multiplying the normalized time series by the total annual cooling demand retrieved from EUROSTAT data^46^.

## Data Records

The developed country-level time series are available in a Zenodo repository^47^ that includes netCDF files for the historical period (1940–2023) created from ERA5 and for climate projections (2006–2100) created with three combinations of global-regional climate models from the CMIP5 EURO-CORDEX and three emission scenarios (RCP 2.6, RCP 4.5 and RCP 8.5). Each country has therefore the following files for the historical period, where ** is the country’s ISO Alpha-2 code:

**__ERA5__ wind__capacity_factor_time_series__onshore.nc
**__ERA5__ wind__capacity_factor_time_series__offshore.nc
**__ERA5__ solar__capacity_factor_time_series.nc
**__ERA5__hydropower__inflow_time_series__conventional_and_pumped_storage.nc
**__ERA5__hydropower__inflow_time_series__run_of_river.nc
**__ERA5__ heating__demand_time_series__residential_space.nc
**__ERA5__ heating__demand_time_series__services_space.nc
**__ERA5__ cooling__demand_time_series.nc
  Each country has also the following files for the climate projections, where ** is the country’s ISO Alpha-2 code, #_# is the value of the RCP scenario (e.g., 2_6 is RCP 2.6), ££££__$$$$ is the name of the combination of global-regional climate models (e.g., CNRM_CERFACS_CM5__CNRM_ALADIN63):
**__CORDEX__RCP_#_#__££££__$$$$__ wind__capacity_factor_time_series__onshore.nc
**__CORDEX__RCP_#_#__££££__$$$$__ wind__capacity_factor_time_series__offshore.nc
**__CORDEX__RCP_#_#__££££__$$$$__ solar__capacity_factor_time_series.nc
**__CORDEX__RCP_#_#__££££__$$$$__hydropower__inflow_time_series__conventional_and_pumped_storage.nc
**__CORDEX__RCP_#_#__££££__$$$$__hydropower__inflow_time_series__run_of_river.nc
**__CORDEX__RCP_#_#__££££__$$$$__ heating__demand_time_series__residential_space.nc
**__CORDEX__RCP_#_#__££££__$$$$__ heating__demand_time_series__services_space.nc
**__CORDEX__RCP_#_#__££££__$$$$__ cooling__demand_time_series.nc

## Technical Validation

The developed time series of the power supply are adjusted with calibration coefficients calculated by comparing simulated generation from today’s installations against generation data retrieved from ENTSO-E’s transparency platform^48^. We used the actual locations of deployed wind turbines, solar photovoltaic panels, and hydropower plants as retrieved from the Open Power System Data (OPSD) platform^49^ and JRC Hydropower Database^42^. By comparing the aggregate generation from actual installations against ENTSO-E data, we ensure that the calibration reflects current conditions, while assuming that the calibration coefficients will remain valid for a potential expanded generation.

The calibration is conducted only for the countries for which data is available, which are: Denmark, France, Germany, Sweden, and the United Kingdom for the onshore wind generation; France, Germany, and the United Kingdom for the solar photovoltaic generation; Austria, Bulgaria, Croatia, France, Greece, Italy, Norway, Portugal, Romania, Serbia, Spain, Sweden, and Switzerland for the conventional and pumped-storage hydropower inflow; and Austria, Croatia, Finland, France, Italy, Portugal, Romania, Serbia, Slovakia and Slovenia for the run-of-river hydropower inflow. The calibration for the onshore wind and solar capacity factors is conducted following the method implemented in Renewable Ninja^15,16^. The hydropower inflow time series is calibrated using the method implemented by Gøtske and Victoria^41^. Details of these calibration procedures are available in the Supplementary Information (Eq. 26–32). For the offshore wind generation, actual generation data at high spatial and temporal resolution against which to perform a calibration is presently not available. Nevertheless, the estimation of wind power generation in offshore regions is expected to be less biased than onshore regions, especially with complex terrain^50^. Note that for both heating and cooling demand we do not perform a calibration as they are strongly correlated with heating and cooling degree days as widely documented in literature^17,44^. While for the cooling demand, actual disaggregated electricity data is lacking, for the heating demand, we show a comparison with demand for natural gas for space heating in the United Kingdom retrieved from its National Transmission System^51^.

**Fig. 3:**
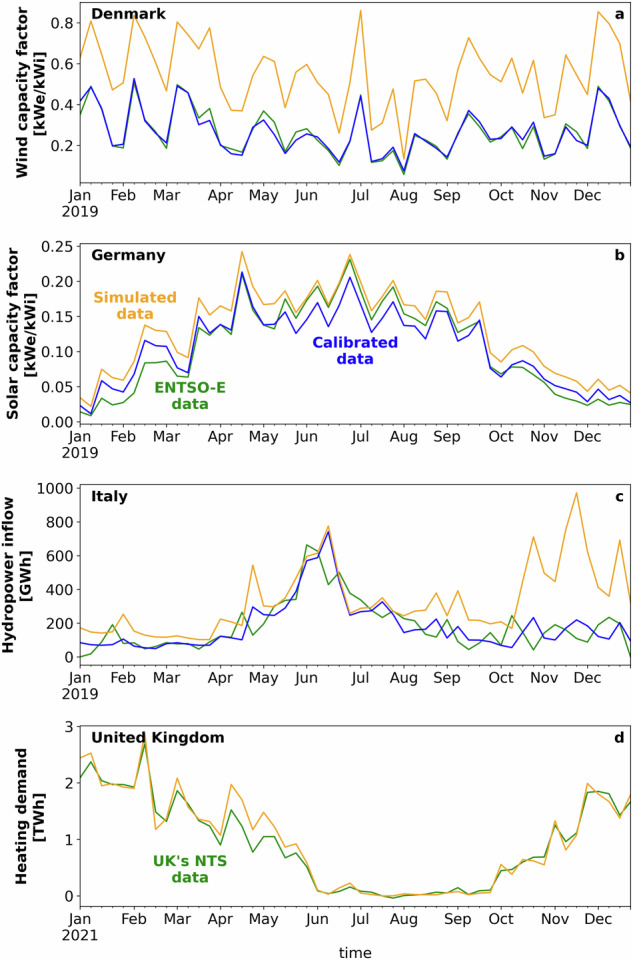
Simulated time series of power supply and demand against actual data. Panel a shows the calibration results for the onshore wind capacity factor for Denmark, panel b shows the calibration results for the solar photovoltaic capacity factor for Germany, panel c shows the calibration results for the conventional and pumped-storage hydropower inflow for Italy, and panel d shows the comparison between actual and simulated space heating demand for the United Kingdom. Results are shown with their weekly values.

In Fig. 3, we show three examples of our calibration procedure and one comparison. The calibration is shown for onshore wind capacity factor in Denmark, solar photovoltaic capacity factor in Germany, conventional and pumped-storage hydropower inflow in Italy, while the comparison is shown for space heating demand in the United Kingdom. Results shown are for one calendar year and when considering ERA5 data as input. Calibration of the wind capacity factor results in a substantial improvement, with a root mean square error decreasing from 32.7 to 6.6. These large discrepancies between the initially simulated capacity factors and the observed ones can be attributed to known biases in the ERA5 data. Reasons for these biases can be identified in the lack of terrain orography, poor coverage of assimilated inputs, and low model resolution^52,53^. The simulated solar photovoltaic capacity factor values are much closer to the observed ones. For wind and solar photovoltaic, unaccounted curtailment—while relatively low (1.5% to 4%) in most large renewable energy markets according to the International Energy Agency data^54^—could also contribute to discrepancies between simulated and observed capacity factors, especially in regions with relatively high penetration of variable renewable energy and lack of effective grid management. The simulated hydropower inflow exhibits good agreement with the observed values from January to October, while it shows larger discrepancies in November and December. These discrepancies can be due to a series of approximations introduced in the hydropower modeling including drainage basin discretization, same hydraulic head, and the lack of accounting for water lost due to evaporation, transpiration, irrigation, or groundwater infiltration. The simulated space heating demand shows relatively good agreement with demand for natural gas indicating the strong correlation between the actual demand and the heating degree days.

**Fig. 4:**
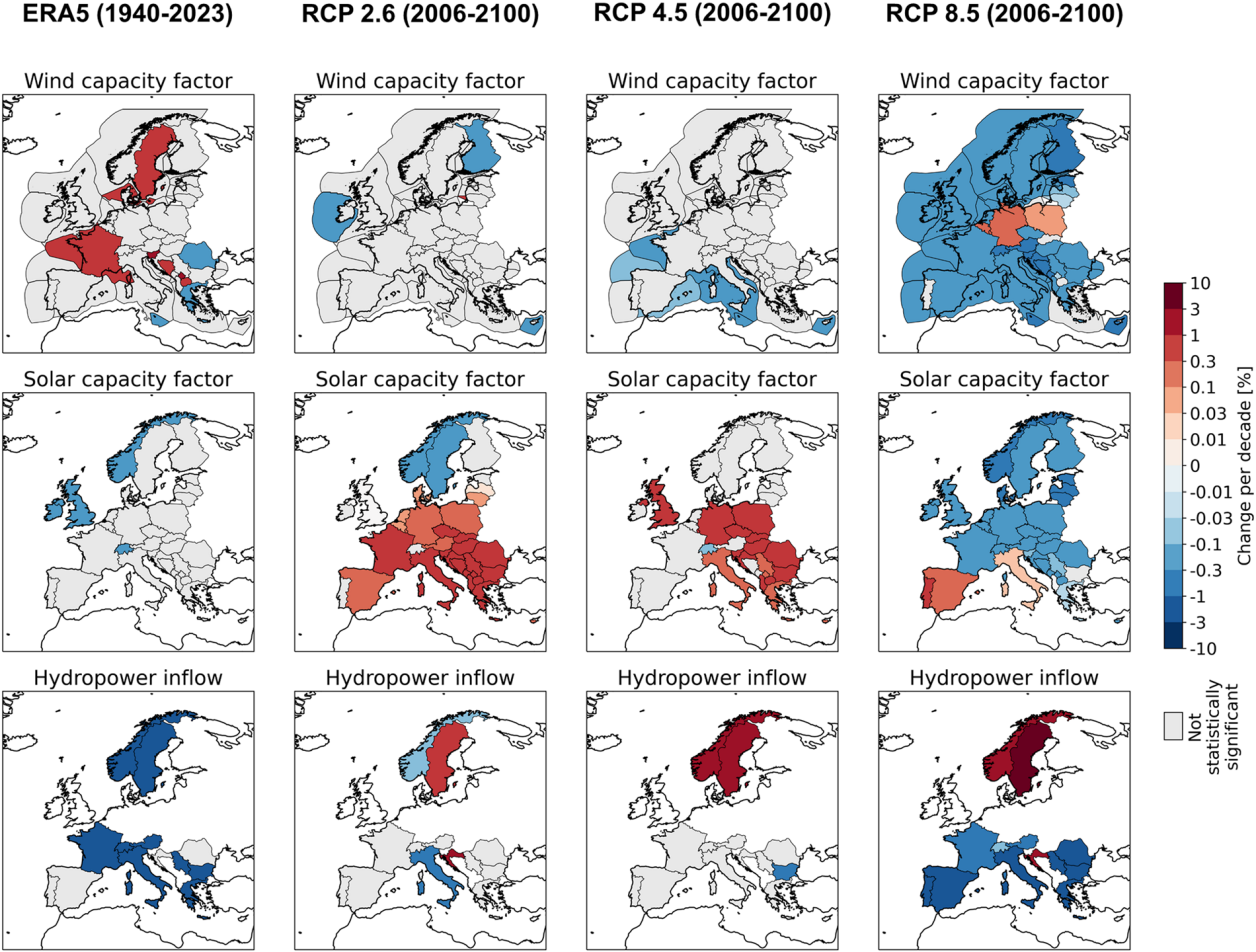
Change in power supply per decade. The panels show changes in onshore and offshore wind capacity factors, solar photovoltaic factors, and conventional and pumped-storage hydropower inflow. We calculated the change per decade with a linear regression of the mean annual values. The columns refer to different meteorological data sources.

Lastly, in Fig. 4, we show an application of our dataset, i.e., the change per decade in power supply for all the countries considered in the analysis. The panels show changes in onshore and offshore wind capacity factors, solar photovoltaic factors, and conventional and pumped-storage hydropower inflow. We calculated the change per decade with a linear regression of the mean annual values. Results indicate that onshore wind does not change significantly for many countries either with historical data or most climate projections. A few positive significant changes are present in the Balkans with historical data whereas negative values are present across Europe for RCP 8.5 climate projections. Results for offshore wind show a broader decrease in capacity factor for most of the countries as the future climate forcing increases. Solar photovoltaic capacity factors show a general increase for RCP2.6 forcing and a tendency to reduced values as the forcing increases. For hydropower, there is a general decrease in inflow for most of the countries with historical meteorological data. Overall, our results show the large heterogeneity and spatial differences in the change of these resources, suggesting that more detailed studies on the impact of climate change on these renewable sources should be conducted at least on a country level and, potentially, at an even finer spatial aggregation, consistently with previous studies^55,56^.

Beside the impact of climate change on power supply, our time series could be used to evaluate the adequacy of power systems largely reliant on these sources in meeting a variable and changing electricity demand. In particular, electricity demand will change as a consequence of the electrification of end-use consumption, such as heating, and the larger adoption of air conditioning, both of which will in turn be affected by a changing climate. The impact on the system adequacy and reliability resulting from these changes can be studied by using the heating and cooling demand time series herein developed. Also, our time series spanning more than 150 years could be used for developing stochastic optimization models to optimize energy system design and operation under uncertain weather and climate conditions; they could be used to investigate robust optimization techniques that can ensure the resilience of energy system designs against a wide range of possible scenarios; or they could be used to evaluate the performance of energy system designs across different possible future scenarios.

## Supplementary information

Supplementary information

## Data Availability

Python scripts that we wrote to process the meteorological data, calculate the country-level time series, and generate the figures are publicly available in the following GitHub repository: https://github.com/eantonini/climapower.

## References

[1] Lund, P. D., Lindgren, J., Mikkola, J. & Salpakari, J. Review of energy system flexibility measures to enable high levels of variable renewable electricity. *Renew. Sustain. Energy Rev.* **45**, 785–807, 10.1016/j.rser.2015.01.057 (2015).

[2] Antonini, E. G. A. *et al*. Identification of reliable locations for wind power generation through a global analysis of wind droughts. *Commun. Earth Environ.* **5**(1), 1–9, 10.1038/s43247-024-01260-7 (2024).

[3] Rinaldi, K. Z., Dowling, J. A., Ruggles, T. H., Caldeira, K. & Lewis, N. S. Wind and Solar Resource Droughts in California Highlight the Benefits of Long-Term Storage and Integration with the Western Interconnect. *Environ. Sci. Technol.* **55**(9), 6214–6226, 10.1021/acs.est.0c07848 (2021). 33822592 10.1021/acs.est.0c07848

[4] Antonini, E. G. A., Ruggles, T. H., Farnham, D. J. & Caldeira, K. Meeting Electricity Demand With Distributed Wind and Solar Generation: System Flexibility Drives Optimal Siting, presented at the ASME 2021 International Mechanical Engineering Congress and Exposition, American Society of Mechanical Engineers Digital Collection, 10.1115/IMECE2021-70678 (2022).

[5] Antonini, E. G. A., Ruggles, T. H., Farnham, D. J. & Caldeira, K. The quantity-quality transition in the value of expanding wind and solar power generation. *iScience* **25**(4), 104140, 10.1016/j.isci.2022.104140 (2022). 35434557 10.1016/j.isci.2022.104140 PMC9010648

[6] Auffhammer, M., Baylis, P. & Hausman, C. H. Climate change is projected to have severe impacts on the frequency and intensity of peak electricity demand across the United States. *Proc. Natl. Acad. Sci.* **114**(8), 1886–1891, 10.1073/pnas.1613193114 (2017). 28167756 10.1073/pnas.1613193114 PMC5338404

[7] van Vliet, M. T. H. *et al*. Vulnerability of US and European electricity supply to climate change, *Nat. Clim. Change*, **2**(9), 9, 10.1038/nclimate1546 (2012).

[8] Schaefli, B. Projecting hydropower production under future climates: a guide for decision-makers and modelers to interpret and design climate change impact assessments. *WIREs Water* **2**(4), 271–289, 10.1002/wat2.1083 (2015).

[9] Bloomfield, H. C. *et al*. Quantifying the sensitivity of european power systems to energy scenarios and climate change projections. *Renew. Energy* **164**, 1062–1075, 10.1016/j.renene.2020.09.125 (2021).

[10] Dubus, L. *et al*. Towards a future-proof climate database for European energy system studies. *Environ. Res. Lett.* **17**(12), 121001, 10.1088/1748-9326/aca1d3 (2022). Nov.

[11] Copernicus Climate Change Service, Climate and energy indicators for Europe from 1979 to present derived from reanalysis, 10.24381/CDS.4BD77450 (2020).

[12] Copernicus Climate Change Service, Climate and energy indicators for Europe from 2005 to 2100 derived from climate projections, 10.24381/CDS.F6951A62 (2021).

[13] Hersbach, H. *et al*. he ERA5 global reanalysis. *Q. J. R. Meteorol. Soc.* **146**(730), 1999–2049, 10.1002/qj.3803 (2020).

[14] CORDEX regional climate model data on single levels: https://cds.climate.copernicus.eu/cdsapp#!/dataset/projections-cordex-domains-single-levels Accessed: Mar. 19 (2024).

[15] Staffell, I. & Pfenninger, S. Using bias-corrected reanalysis to simulate current and future wind power output. *Energy* **114**, 1224–1239, 10.1016/j.energy.2016.08.068 (2016).

[16] Pfenninger, S. & Staffell, I. Long-term patterns of European PV output using 30 years of validated hourly reanalysis and satellite data. *Energy* **114**, 1251–1265, 10.1016/j.energy.2016.08.060 (2016).

[17] Staffell, I., Pfenninger, S. & Johnson, N. A global model of hourly space heating and cooling demand at multiple spatial scales. *Nat. Energy* **8**(12), 1328–1344, 10.1038/s41560-023-01341-5 (2023).

[18] Gelaro, R. *et al*. The Modern-Era Retrospective Analysis for Research and Applications, Version 2 (MERRA-2). *J. Clim.* **30**(14), 5419–5454, 10.1175/JCLI-D-16-0758.1 (2017). 10.1175/JCLI-D-16-0758.1 PMC6999672 32020988

[19] Bloomfield, H. C., Brayshaw, D. J., Deakin, M. & Greenwood, D. Hourly historical and near-future weather and climate variables for energy system modelling. *Earth Syst. Sci. Data* **14**(6), 2749–2766, 10.5194/essd-14-2749-2022 (2022).

[20] PRIMAVERA: https://www.primavera-h2020.eu/ Accessed: Mar. 19 (2024).

[21] Formayer, H. *et al*. SECURES-Met: A European meteorological data set suitable for electricity modelling applications. *Sci. Data* **10**(1), 590, 10.1038/s41597-023-02494-4 (2023). 37679367 10.1038/s41597-023-02494-4 PMC10484998

[22] Buster, G., Benton, B. N., Glaws, A. & King, R. N. High-resolution meteorology with climate change impacts from global climate model data using generative machine learning, *Nat. Energy*, pp. 1–13, 10.1038/s41560-024-01507-9 (2024).

[23] Ruggles, T. H. *et al*. Planning reliable wind- and solar-based electricity systems. *Adv. Appl. Energy* **15**, 100185, 10.1016/j.adapen.2024.100185 (2024).

[24] Protected Areas (WDPA), Protected Planet: https://www.protectedplanet.net/en/thematic-areas/wdpa Apr. 09, (2024).

[25] CORINE Land Cover: https://land.copernicus.eu/en/products/corine-land-cover Accessed: Apr. 09, (2024).

[26] Schiavina, M., Freire, S., Carioli, A. & MacManus, K. GHS-POP R2023A - GHS population grid multitemporal (1975–2030). European Commission, Joint Research Centre (JRC), 10.2905/2FF68A52-5B5B-4A22-8F40-C41DA8332CFE, (2023).

[27] Hofmann, F., Hampp, J., Neumann, F., Brown, T. & Hörsch, J. atlite: A Lightweight Python Package for Calculating Renewable Power Potentials and Time Series. *J. Open Source Softw.* **6**(62), 3294, 10.21105/joss.03294 (2021).

[28] Taylor, K. E., Stouffer, R. J. & Meehl, G. A. An Overview of CMIP5 and the Experiment Design. *Bull. Am. Meteorol. Soc.* **93**(4), 485–498, 10.1175/BAMS-D-11-00094.1 (2012).

[29] Voldoire, A. *et al*. The CNRM-CM5.1 global climate model: description and basic evaluation. *Clim. Dyn.* **40**(9), 2091–2121, 10.1007/s00382-011-1259-y (2013).

[30] Giorgetta, M. A. *et al*. Climate and carbon cycle changes from 1850 to 2100 in MPI-ESM simulations for the Coupled Model Intercomparison Project phase 5. *J. Adv. Model. Earth Syst.* **5**(3), 572–597, 10.1002/jame.20038 (2013).

[31] Jones, C. D. *et al*. The HadGEM2-ES implementation of CMIP5 centennial simulations. *Geosci. Model Dev.* **4**(3), 543–570, 10.5194/gmd-4-543-2011 (2011).

[32] Abdel-Lathif, A. Y., Roehrig, R., Beau, I. & Douville, H. Single-Column Modeling of Convection During the CINDY2011/DYNAMO Field Campaign With the CNRM Climate Model Version 6. *J. Adv. Model. Earth Syst.* **10**(3), 578–602, 10.1002/2017MS001077 (2018).

[33] Sitz, L. E. *et al*. Description and evaluation of the Earth System Regional Climate Model (Reg CM-ES). *J. Adv. Model. Earth Syst.* **9**(4), 1863–1886, 10.1002/2017MS000933 (2017).

[34] Rockel, B., Will, A. & Hense, A. The Regional Climate Model COSMO-CLM (CCLM), *Meteorol. Z*. pp. 347–348, 10.1127/0941-2948/2008/0309 (2008).

[35] Cucchi, M. *et al*. WFDE5: bias-adjusted ERA5 reanalysis data for impact studies. *Earth Syst. Sci. Data* **12**(3), 2097–2120, 10.5194/essd-12-2097-2020 (2020).

[36] *IEAWindTask37/IEA-3.4-130-RWT*. F*. IEAWindTask37: https://github.com/IEAWindTask37/IEA-3.4-130-RWT Accessed: Feb. 22, 2024 (2023).

[37] *IEAWindTask37/IEA-10.0-198-RWT*. Python. IEAWindTask37: https://github.com/IEAWindTask37/IEA-10.0-198-RWT Accessed: Feb. 22, 2024 (2024).

[38] Bortolotti, P. *et al*. IEA Wind TCP Task 37: Systems Engineering in Wind Energy - WP2.1 Reference Wind Turbines, National Renewable Energy Laboratory, NREL/TP-5000-73492.

[39] Stephenson, P. J. Maritime Spatial Planning in Europe. Discussion Paper on the Challenges and Potential Opportunities Around the Colocation of Offshore Wind Energy with Marine Protected Areas, Renewables Grid Initiative, Berlin, Germany (2023).

[40] Antonini, E. G. A. & Caldeira, K. Spatial constraints in large-scale expansion of wind power plants. *Proc. Natl. Acad. Sci.* **118**(27), e2103875118, 10.1073/pnas.2103875118 (2021). 34183400 10.1073/pnas.2103875118 PMC8271749

[41] Gøtske, E. K. & Victoria, M. Future operation of hydropower in Europe under high renewable penetration and climate change. *iScience* **24**(9), 102999, 10.1016/j.isci.2021.102999 (2021). 34505010 10.1016/j.isci.2021.102999 PMC8413898

[42] JRC, *energy-modelling-toolkit/hydro-power-database*. Energy Modelling Toolkit. https://github.com/energy-modelling-toolkit/hydro-power-database Accessed: 04, 2024 (2024).

[43] HydroBASINS: https://www.hydrosheds.org/products/hydrobasins (2024).

[44] Ruhnau, O., Hirth, L. & Praktiknjo, A. Time series of heat demand and heat pump efficiency for energy system modeling. *Sci. Data* **6**(1), 189, 10.1038/s41597-019-0199-y (2019). 31575870 10.1038/s41597-019-0199-y PMC6773861

[45] Ashfaq, A. & Ianakiev, A. Cost-minimised design of a highly renewable heating network for fossil-free future. *Energy* **152**, 613–626, 10.1016/j.energy.2018.03.155 (2018).

[46] Database - Eurostat: https://ec.europa.eu/eurostat/data/database (2024).

[47] Antonini, E. G. A., Di Bella, A., Drouet, L., Savelli, I. & Tavoni, M. Weather- and climate-driven power supply and demand time series for European countries. *Zenodo*10.5281/zenodo.13938926 (2024). 10.1038/s41597-024-04129-8 PMC11618340 39632853

[48] ENTSO-E Transparency Platform: https://transparency.entsoe.eu/ (2024).

[49] Open Power System Data – A platform for open data of the European power system: https://open-power-system-data.org/ (2024).

[50] Gualtieri, G. Analysing the uncertainties of reanalysis data used for wind resource assessment: A critical review. *Renew. Sustain. Energy Rev.* **167**, 112741, 10.1016/j.rser.2022.112741 (2022).

[51] National Gas Transmission Data Portal: https://data.nationalgas.com/find-gas-data (2024).

[52] Rose, S. & Apt, J. What can reanalysis data tell us about wind power? *Renew. Energy* **83**, 963–969, 10.1016/j.renene.2015.05.027 (2015).

[53] Davidson, M. R. & Millstein, D. Limitations of reanalysis data for wind power applications. *Wind Energy* **25**(9), 1646–1653, 10.1002/we.2759 (2022).

[54] Will more wind and solar PV capacity lead to more generation curtailment? – Renewable Energy Market Update - 2023 – Analysis, IEA: https://www.iea.org/reports/renewable-energy-market-update-june-2023/will-more-wind-and-solar-pv-capacity-lead-to-more-generation-curtailment (2024).

[55] Gernaat, D. E. H. J. *et al*. Climate change impacts on renewable energy supply. *Nat. Clim. Change* **11**(2), 119–125, 10.1038/s41558-020-00949-9 (2021).

[56] Liu, L. *et al*. Climate change impacts on planned supply–demand match in global wind and solar energy systems, *Nat. Energy* **8**, 8, 10.1038/s41560-023-01304-w (2023).

