# Supplementary material for: Weather- and climate-driven power supply and demand time series for power and energy system analyses
Source: Sci Data. 2024 Dec 4;11:1324. doi: 10.1038/s41597-024-04129-8 (PMC11618340; doi:10.1038/s41597-024-04129-8)
Supplement: Supplementary file 1 — Supplementary information [file 41597_2024_4129_MOESM1_ESM.pdf]

Supplementary information for

Weather- and climate-driven power supply and demand time series for power and energy system analyses

Authors: Enrico G. A. Antonini[a,b], Alice Di Bella[a,b,c], Iacopo Savelli[a,d], Laurent Drouet[a,b], Massimo Tavoni[,a,b,c]

Affiliation:

a) CMCC Foundation - Euro-Mediterranean Center on Climate Change, Lecce, Puglia, Italy

b) RFF-CMCC European Institute on Economics and the Environment, Milan, Lombardy, Italy

c) Politecnico di Milano, Milan, Lombardy, Italy

d) Università Bocconi, Milan, Lombardy, Italy

Corresponding author: Enrico G. A. Antonini, enrico.antonini@cmcc.it

		C3S [11], [12]	Renewable and Demand Ninja [15], [16], [17]	Bloomfield et al. [19]	SECURES-Met [21]	Buster et al. [22]	Ours
**Spatial scope**		Europe	Global	Europe	Europe	Global	Europe
**Climate data source**	**Historical**	ERA5	MERRA-2	ERA5	ERA5		ERA5
	**Projections**	CMIP5 EURO-CORDEX		ERA5 + PRIMAVERA	CMIP5 EURO-CORDEX	CMIP6	CMIP5 EURO-CORDEX
**Supply**	**Wind**	Wind speed and capacity factor	Wind capacity factor	Wind speed and capacity factor	Wind capacity factor	Wind speed	Wind capacity factor
	**Solar**	Solar radiation and capacity factor	Solar capacity factor	Solar radiation and capacity factor	Solar radiation	Solar radiation	Solar capacity factor
	**Hydropower**	Hydropower generation			Hydropower generation		Hydropower inflow
**Demand**	**Heating**		Heating demand	Heating degree days			Heating demand
	**Cooling**		Cooling demand	Cooling degree days			Cooling demand

Supplementary Table 1. Datasets providing weather- and climate-driven power supply and demand time series for power and energy system analysis. We show a comparison between the dataset that we developed (on the rightmost column) and other available energy datasets.

Wind power generation

For each grid cell of the selected dataset, we calculate the time series of the wind power generation. This depends on the wind speed at the turbine hub height and the chosen turbine, which is characterized by a power curve. Here, we consider two wind turbines, the IEA-3.4-130-RWT [36] for onshore and IEA-10.0-198-RWT [37] for offshore installations. These wind turbines were developed within the IEA Wind Task 37 [38]. Other turbines can be chosen from the ones that are implemented in Atlite or new turbines can easily be defined. First, we calculate the wind speed, V, in case its two components, V_x and V_y, are provided:

$$V = \sqrt{V_x^2 + V_y^2}. \tag{1}$$

Second, the wind speed of the selected dataset at the given height, $V(z_{dataset})$, is extrapolated to the turbine hub height, $V(z_{turbine})$, using a well-established logarithmic function [S1]:

$$V(z_{turbine}) = V(z_{dataset}) \frac{\ln(z_{turbine}/z_0)}{\ln(z_{dataset}/z_0)}, \tag{2}$$

where z_0 is the terrain surface roughness. Lastly, the resulting wind speed is used to get the power generation from the wind turbine power curve, f:

$$P = f(V). \tag{3}$$

The extrapolation and conversion operations are performed using Atlite's built-in functionalities.

To calculate the country-level time series of the wind capacity factor, we perform an aggregation using the available area with the wind power in the first quartile. For onshore applications, from the country's available land, we subtract all protected areas of the World Database on Protected Areas (WDPA), as well as areas within 500 m from urban, industrial, and commercial centers. For offshore applications, from the country's exclusive economic zone, we remove protected areas and consider only water within 100 km from the coast. Of the resulting available area, we consider only the fraction with the resources in the first quartile. This area for potential installations allows room for other socio-economic constraints, which are not considered in this methodology, while preserving more diversified wind resources. We consider the area for

potential installations because currently installed capacity is still a marginal fraction in the supply mix and because locations of installed capacity are typically not available.

Solar photovoltaics power generation

For each grid cell of the selected dataset, we calculate the time series of the solar photovoltaics power generation. This depends on the time of the day, position on the Earth, solar irradiance, and panel efficiency. First, we calculate the approximate solar coordinates to an accuracy of about 1 arcminute within two centuries of 2000 [S2], [S3]. The algorithm requires the Julian date, JD, of the time for which the Sun's coordinates are needed. The first step in this approximation is to compute the number of days and fraction, n, from noon of January 1, 2000, or Julian date 2451545:

$$n = JD - 2451545. \tag{4}$$

The second step is the calculation of the mean longitude, L, and mean anomaly, g, of the Sun in degrees:

$$L = 280.460 + 0.9856474\, n, \tag{5}$$

$$g = 357.528 + 0.9856003\, n. \tag{6}$$

The Sun's ecliptic longitude, λ, in degrees can be approximated as:

$$\lambda = L + 1.915 \sin(g) + 0.020 \sin(2g)\,, \tag{7}$$

while the ecliptic latitude can be approximated to zero $(\beta = 0)$. The mean obliquity of the ecliptic, ε, in degrees can be approximated as:

$$\varepsilon = 23.439 - 3.6 \cdot 10^{-7}\, n. \tag{8}$$

The Sun's right ascension, RA, and declination angle, δ, can be obtained from:

$$RA = \tan^{-1}\left[\frac{\cos(\varepsilon)\sin(\lambda)}{\cos(\lambda)}\right], \tag{9}$$

$$\delta = \sin^{-1}[\sin(\lambda)\sin(\varepsilon)]\,. \tag{10}$$

The local mean sidereal time can be approximated as:

$$\text{lmst} \ = \ 15\ \left[6.697375 + \left(\text{hour} + \frac{\text{minute}}{60}\right) + 0.0657098242\ n\right] + \varphi, \tag{11}$$

where φ is the longitude. Lastly, given the hour angle, ω:

$$\omega = \text{lmst}\ - RA, \tag{12}$$

the Sun's altitude, α_S, and the azimuth, γ_S, can be calculated as follows:

$$\alpha_S = \sin^{-1}[\cos(\delta)\cos(\phi)\cos(\omega) + \sin(\delta)\sin(\omega)], \tag{13}$$

$$\gamma_S = \cos^{-1}\left[\frac{\sin(\delta)\cos(\phi) - \cos(\delta)\sin(\delta)\sin(\omega)}{\cos(\alpha_S)}\right], \tag{14}$$

where ϕ is the latitude.

Given the solar position, the angle of incidence of the solar radiation on a tilted surface is given by:

$$i = \cos^{-1}[\sin(\beta)\sin(\alpha_S)\cos(\gamma_S - \gamma_P) + \cos(\alpha_S)\cos(\beta)]\,, \tag{15}$$

where γ_P and β are the surface's azimuth and tilt angles, respectively.

The total radiation received by a tilted surface is composed of three components: direct, diffuse, and reflected radiation. To calculate the diffuse radiation, several models are available. Here we use the Reindl sky diffuse irradiance model [S4]. Lastly, given the solar radiation on a horizontal surface,

$$I_h = I_{b,h} + I_{d,h}, \tag{16}$$

where $I_{b,h}$ and $I_{d,h}$ are the direct and diffuse radiation on a horizontal surface, the solar radiation incident on a tilted panel can be calculated as:

$$I_P = I_{b,h}\frac{\cos(i)}{\sin\alpha_S} + I_{d,h}\frac{1+\cos(\beta)}{2} + \rho I_h\frac{1-\cos(\beta)}{2}, \quad (17)$$

where ρ is the surface's albedo used to calculate the reflected component.

Lastly, the solar photovoltaics power generation can be calculated from the solar panel power curve, f, which depends on the incident solar radiation and ambient temperature, t_a:

$$P = f(I_P, t_a). \quad (18)$$

The calculation of the Sun's position, the incidence angle, the incidence radiation, and the power conversion are performed using Atlite's built-in functionalities. To calculate the country-level time series of the solar photovoltaics capacity factor, we perform an aggregation using the available area with the resources in the first quartile, in a way similar to the wind power capacity factor.

Hydropower inflow

For each hydropower plant and for each dataset, we calculate the time series of the inflow following the methodology developed by Gøtske and Victoria [41]. First, from the location of the currently installed hydropower plants (JRC Hydropower Database [42]) and hydrological maps (HydroBASINS [43] with level 8 basins delineation), we identify the grid cells that belong to each plant's upstream drainage basins. We aggregate runoff values, RO_{cell}, in each basin to determine the basin inflow, Q_{basin}, and multiply the resulting value by its basin surface, A_{basin}, and water density (if ERA5 data are used) or time step resolution (if CORDEX data are used), resulting in a unit of kg per time step:

$$Q_{\text{basin}} = A_{\text{basin}}\left(\sum_{\text{cells}\in\text{basin}} RO_{\text{cell}}\right). \quad (19)$$

Using Atlite's built-in functionality, the inflow to any hydropower power plant is calculated by considering a water flow speed, v_{RO} (assumed speed equal to 1 m/s), from any upstream basin to its reservoir, whose distance is defined as L_{basin}, and aggregating their values:

$$Q_{\text{plant}}(t) = \sum_{\substack{\text{upstream} \\ \text{basins}}} Q_{\text{basin}}\left(t - \frac{L_{\text{basin}}}{v_{RO}}\right). \tag{20}$$

To estimate the hydropower inflow in unit of energy, in the absence of available individual plant hydraulic head, we assume a mean value of 50 m, h, for all the power plants when considering conventional and pumped-storage hydropower and a value of 10 m when considering run of river hydropower:

$$E_{\text{plant}} = ghQ_{\text{plant}}, \tag{21}$$

where g is the gravitational acceleration. The country-level hydropower inflow is obtained by summing up the inflow of all hydropower plants.

Heating demand

For each country and for each dataset, we calculate the time series of the space heating demand. This depends on the heating degree days, the intraday heating demand, and the population density. First, from the hourly time series of the temperature in each grid cell, we calculate the heating degree days, HDD, i.e., the difference between the daily mean air temperature and the lowest daily mean air temperature not leading to indoor heating, $T_{\text{ref},HDD}$:

$$HDD = \begin{cases} T_{\text{ref},HDD} - T_{\text{daily mean}} & \text{if } T_{\text{daily mean}} < T_{\text{ref},HDD} \\ 0 & \text{if } T_{\text{daily mean}} \geq T_{\text{ref},HDD} \end{cases}. \tag{22}$$

We use a value of $T_{\text{ref},HDD}$ equal to 15 °C. We then aggregate the grid-cell-level time series of the heating degree days by performing a weighted average using the population density in each grid cell to obtain a country-level time series.

Second, we calculate normalized intraday hourly heating demand profiles, i.e., the heating demand over 24 hours, following the method developed by Ruhnau et al. [44]. In summary, intraday heating profiles that depend on daily mean temperature, hour of the day, and day of the week are taken from the Bundesverband der Energie- und Wasserwirtschaft (BDEW) (German Association of Energy and Water Industries). Different profiles are available for residential and commercial buildings. These profiles can be interpreted as hourly shares of the daily demand.

We then build an hourly heating demand time series by concatenating these intraday profiles multiplied by the respective heating degree days. Lastly, the time series is normalized by its time integral, such that the actual heating demand time series can be obtained by multiplying the normalized time series by the total annual heating demand retrieved from EUROSTAT data [46].

Cooling demand

For each country and for each dataset, we calculate the time series of the space cooling demand. This depends on the cooling degree days, the intraday cooling demand, and the population density. First, from the hourly time series of the temperature in each grid cell, we calculate the cooling degree days, CDD, i.e., the difference between the daily mean air temperature and the highest daily mean air temperature not leading to indoor cooling, $T_{ref,CDD}$:

$$CDD = \begin{cases} T_{\text{daily mean}} - T_{ref,CDD} & \text{if } T_{\text{daily mean}} > T_{\text{ref},CDD} \\ 0 & \text{if } T_{\text{daily mean}} \leq T_{\text{ref},CDD} \end{cases}. \quad (23)$$

We use a value of $T_{ref,CDD}$ equal to 24 °C. We then aggregate the grid-cell-level time series of the cooling degree days by performing a weighted average using the population density in each grid cell to obtain a country-level time series.

Second, due to unavailability of actual electricity data at high spatial and temporal resolution that isolate the cooling demand, we build an intraday cooling demand profile. We calculate cooling degree hours, CDH, i.e., the difference between the hourly air temperature and the highest hourly air temperature not leading to indoor cooling:

$$CDH = \begin{cases} T_{\text{hourly}} - T_{\text{ref},CDH} & \text{if } T_{\text{hourly}} > T_{\text{ref},CDH} \text{ and } CDD > 0 \\ 0 & \text{if } T_{\text{hourly}} \leq T_{\text{ref},CDH} \end{cases}. \quad (24)$$

We use a value of $T_{\text{ref},CDH}$ equal to 28 °C. We keep the values of the cooling degree hours only on the days where the cooling degree days are positive. This approach implies that short excursions above the cooling threshold temperature would not increase internal temperatures sufficiently to require cooling. We then again perform an aggregation using the population density. Lastly, the time series is normalized by its time integral, such that the actual cooling

demand time series can be obtained by multiplying the normalized time series by the total annual cooling demand retrieved from EUROSTAT data [46].

Aggregation procedure

To calculate the country-level time series from the gridded time series, we perform an aggregation. This is a weighted average, where the weights are the gridded values of the aggregation variables. The aggregation variables are the fraction of available land in a grid cell when considering wind and solar generation, the fraction of grid cell belonging to a hydrological basin when considering hydropower generation, and the population in a grid cell when considering heating and cooling demand. The weighted average is performed according to the following equation:

$$\text{time\_series}(t) = \frac{\sum_{i\in \text{grid\_cells}} \text{time\_series}(t, i)\ \text{variable}(i)}{\sum_{i\in \text{grid cells}} \text{variable}(i)}. \qquad (25)$$

Power supply calibration

The calibration for wind and solar capacity factors is conducted following the method implemented in Renewable Ninja [15], [16]. In summary, the wind speed at the turbine hub height derived from the climate model data is corrected using a multiplicative factor (α) and a linear offset (β):

$$V' = \alpha V + \beta. \qquad (26)$$

The multiplicative factor is given by:

$$\alpha = 0.6 \frac{CF_{\text{mean,obs}}}{CF_{\text{mean,sim}}} + 0.2, \qquad (27)$$

where $CF_{\text{mean,obs}}$ $CF_{\text{mean,sim}}$ are the observed and simulated country-level capacity factors. The observed capacity factor is calculated by dividing the generation by the installed capacity retrieved from ENTSO-E. The simulated country-level capacity factor is obtained by aggregating

grid-level capacity factors where turbines are located. The linear offset is found by an iterative search to minimize the bias between the observed and simulated capacity factors:

$$\begin{aligned} \underset{\beta}{\text{minimize}} \;\; & |CF_{\text{obs}} - CF_{\text{sim}}'| \\ \text{subject to} \;\; & \beta \geq 0 \end{aligned} . \tag{28}$$

For solar, we perform a similar operation where the simulated country-level capacity factor is again corrected using a multiplicative factor (α) and a linear offset (β):

$$CF_{\text{sim}}' = \alpha CF_{\text{sim}} + \beta. \tag{29}$$

The observed capacity factor is calculated by dividing the generation by installed capacity retrieved from ENTSO-E. The simulated country-level capacity factor is obtained by aggregating grid-level capacity factors where solar panels are located. The two correction factors are found with an iterative search to minimize the bias between the observed and simulated capacity factors:

$$\begin{aligned} \underset{\alpha,\beta}{\text{minimize}} \;\; & |CF_{\text{obs}} - CF_{\text{sim}}'| \\ \text{subject to} \;\; & \begin{matrix} 0 \leq \alpha \leq 2 \\ -0.5 \leq \beta \leq 0.5 \end{matrix} \end{aligned} . \tag{30}$$

The hydropower inflow time series is calibrated using the method implemented by Gøtske and Victoria [41]. For conventional and pumped-storage hydropower, the observed weekly hydropower inflow, I_w, is calculated by considering the reservoir level between two weeks, R_w and R_{w-1} (expressed in unit of energy), and the generation over that week, G_{w-1}:

$$I_w = R_w - R_{w-1} + G_{w-1}. \tag{31}$$

For each month of the year, we then calculate retain factors, or calibration coefficients, which account for the level of water lost due to evaporation, transpiration, irrigation, or groundwater infiltration before reaching the reservoir. These are calculated as the ratio between the observed inflow and the simulated inflow:

$$RF_m = \frac{I_{m,\text{obs}}}{I_{m,\text{sim}}} \tag{32}$$

For run-of-river hydropower, we calibrate the simulated inflow against actual generation as such power plants do not have water storage capacity.

Supplementary references

[S1] G. B. Andresen, A. A. Søndergaard, and M. Greiner, “Validation of Danish wind time series from a new global renewable energy atlas for energy system analysis,” *Energy*, vol. 93, pp. 1074–1088, Dec. 2015, doi: 10.1016/j.energy.2015.09.071.

[S2] J. J. Michalsky, “*The Astronomical Almanac*’s algorithm for approximate solar position (1950–2050),” *Solar Energy*, vol. 40, no. 3, pp. 227–235, Jan. 1988, doi: 10.1016/0038-092X(88)90045-X.

[S3] A. B. Sproul, “Derivation of the solar geometric relationships using vector analysis,” *Renewable Energy*, vol. 32, no. 7, pp. 1187–1205, Jun. 2007, doi: 10.1016/j.renene.2006.05.001.

[S4] D. T. Reindl, W. A. Beckman, and J. A. Duffie, “Diffuse fraction correlations,” *Solar Energy*, vol. 45, no. 1, pp. 1–7, Jan. 1990, doi: 10.1016/0038-092X(90)90060-P.
